# Phytosanitary Irradiation

**DOI:** 10.3390/foods5010008

**Published:** 2016-01-20

**Authors:** Guy J. Hallman, Carl M. Blackburn

**Affiliations:** Joint FAO/IAEA Programme on Nuclear Techniques in Food and Agriculture, P.O. Box 100, A-1400 Vienna, Austria; c.m.blackburn@iaea.org

**Keywords:** phytosanitary treatment, food irradiation, insects, pests, fruits

## Abstract

Phytosanitary treatments disinfest traded commodities of potential quarantine pests. Phytosanitary irradiation (PI) treatments use ionizing radiation to accomplish this, and, since their international commercial debut in 2004, the use of this technology has increased by ~10% annually. Generic PI treatments (one dose is used for a group of pests and/or commodities, although not all have been tested for efficacy) are used in virtually all commercial PI treatments, and new generic PI doses are proposed, such as 300 Gy, for all insects except pupae and adult Lepidoptera (moths). Fresh fruits and vegetables tolerate PI better than any other broadly used treatment. Advances that would help facilitate the use of PI include streamlining the approval process, making the technology more accessible to potential users, lowering doses and broadening their coverage, and solving potential issues related to factors that might affect efficacy.

## 1. Introduction

Foods, such as fresh fruits and vegetables, which are transported between countries and regions, may harbor invasive pests that could become established in new areas, harming crops, the environment, livelihoods, and economies. Pest risk assessments and commensurate restrictions on the movements of fresh produce are used to prevent invasive species from spreading. Without effective phytosanitary measures, fresh fruits and vegetables from affected regions can be prohibited from crossing quarantine boundaries within or between countries.

Phytosanitary measures comprise legislation, regulation, or official procedure having the purpose to prevent the introduction or spread of quarantine pests or to limit the economic impact of regulated non-quarantine pests. Treatments are phytosanitary measures designed to kill, inactivate, remove, or render phytosanitary pests infertile and must be certified to a degree of efficacy near 100% in order to permit trade in otherwise quarantined items while preventing the spread of viable pests.

Insects most often raising phytosanitary concerns in terms of trade in fresh produce are, in decreasing order of global importance, fruit flies (family Tephritidae, e.g., Mediterranean fruit fly); butterflies and moths (Order Lepidoptera, e.g., codling moth, oriental fruit moth), and; mealybugs (family Pseudococcidae) [1]. Other important regulated pest groups are scale insects, weevils, whiteflies, thrips, and mites.

Commercial phytosanitary treatments include cold (~0–2 °C), heat (~44–48 °C), fumigation (methyl bromide, phosphine), and, increasingly in recent years, ionizing radiation [2]. Ionizing radiation is used as a phytosanitary treatment in a growing number of countries for an increasing number of fruits and vegetables against a growing number of different pests [1]. The commercial use of phytosanitary irradiation has increased by ~10% every year since 2000 as the use of chemical fumigants is restricted, irradiation treatment protocols and their applicability to different fruits and vegetables are accepted internationally, and more countries and traders adopt the procedure for intra- and inter-national shipments.

## 2. Nature of Ionizing Radiation

Radiation is the emission of energy as waves (e.g., X-rays) or as moving particles (e.g., electron beams), and it is classified as ionizing if it has sufficient energy to remove electrons from atoms or molecules, for example to create an ion. The definition makes it difficult to precisely describe the energy region at which radiation becomes considered as ionizing since there are many different molecules and atoms and they can be ionized at a broad range of different energies. For example although visible light is generally considered as non-ionizing it ionizes certain chemicals, such as chlorophyll, which is ionized to initiate photosynthesis. Ultraviolet light, which is shorter in wavelength (10 to 400 nm with photon energies of 3 to 124 eV) than visible light can cause ionization and has been studied as a phytosanitary treatment for surface pests [1,2]. Ionizing radiation is generally considered to comprise radiation with energy of several hundreds of kiloelectronvolts or above, and, therefore, includes the higher frequency portion of the electromagnetic spectrum, such as X-rays and gamma rays or high-energy particles, such as electron beams. Gamma rays from the isotope cobalt-60 (1.17 and 1.33 MeV) or caesium-137 (0.66 MeV), electron beams, or X-rays may be used for food irradiation according to the Codex Alimentarius Commission (CAC) [3]. Cobalt-60 is produced from non-radioactive cobalt via neutron irradiation and has a half-life of 5.27 years. When cobalt-60 decays it emits a beta particle and in doing so becomes nickel in one of two highly energetic states, and in actual fact it is this nickel that promptly emits gamma radiation. Caesium-137 has been used for research into food irradiation. It is a nuclear fission product, recovered when processing spent nuclear fuel, has a half-life of 30.07 years, and decays to barium-137 m. Although its longer half-life make it more attractive as an irradiation source than cobalt-60, caesium-137 is not used to irradiate food commercially because cobalt-60 offers higher radiation energy output for a given volume and is also chemically stable in the metallic form. In contrast, caesium is ionic, the stable forms are water soluble salts (e.g., caesium chloride), and the caesium ion is mobile in the environment.

Machine sources of ionizing radiation are electron beams (e-beams) and X-rays. E-beams can be used for food irradiation at energy levels up to 10 MeV. An e-beam directed at a heavy metal (e.g., tantalum or gold) emits X-rays, and energies up to 5.0 and 7.5 MeV, respectively, are allowed for food irradiation by the CAC [3] and the United States Food and Drug Administration [4]. At best, ~14% of the energy from an e-beam is converted to X-rays with the rest lost as heat. The energy of these beams or rays does not induce measurable amounts of radioactivity in food [5,6].

Gamma and X-rays are electromagnetic and have zero rest mass; therefore, they penetrate through large bulky consignments, such as pallets of fruits and vegetables and into any insects that might be present causing tracks with spurs of ionization events along their path. Electron beams are particulate in nature and cause ionization events in dense clusters transferring their energy rapidly. They cannot penetrate as deeply as rays, and are applied to relatively small dimensions of fruits and vegetables (~20–30 cm) often via a two pass irradiation treatment (irradiating first from one side of the package and then the opposite side). Gamma and X-rays, with greater penetration, can be used to treat entire pallet loads of packed product, generally using multiple passes of the radiation source to improve the dose distribution where the containers are irradiated from at least two opposite sides. However, the transfer of energy from gamma ray, X-ray, or electron beam is broadly similar; either the radiation directly removes an electron from a biologically important chemical (e.g., DNA) or electrons are liberated from chemicals in the bulk of the target material and the resulting free electrons, ions, and free radicals react further with their surroundings, ultimately damaging biologically important molecules. Thus, the radiation either directly or indirectly disrupts the structure of organic molecules in the insect, and, above a specific radiation dosage, the disruption is sufficient to prevent insects from developing further or reproducing. Simply because DNA is such a huge target on the molecular level and is key to initiating growth and reproduction, damage to it is generally the reason pests are controlled via irradiation.

## 3. History and Current Use of Ionizing Radiation as a Quarantine Treatment

Hallman [1] discusses the history of phytosanitary irradiation (PI); this current section provides clarifications and more recent developments. Research in the early 1900s demonstrated the effectiveness of X-rays against the development of egg, larva, and adult cigar beetle to prevent damage to cigars [7]. The first notion of using ionizing radiation as a phytosanitary treatment was published by Koidsumi [8] who suggested it to disinfest fruit of tephritid fruit flies. He also noted that, unlike all other phytosanitary treatments, acute mortality was not necessary to provide quarantine security, but that prevention of adult emergence was a reasonable objective, and this is the measurement of efficacy used today in the internationally accepted generic PI dose for tephritid fruit flies [9].

In 1972, the US state of Hawaii petitioned the US Food and Drug Administration (FDA) to use PI on papayas [10], and 14 years later the FDA [4] approved the use of up to 1 kGy irradiation to disinfest foods of arthropods. That same year (1986), the first commercial use of PI occurred when one load of irradiated mangoes was shipped from Puerto Rico for sale in Florida [2]. The next year a consignment of irradiated Hawaiian papayas was shipped to California, and in 1989 the Animal and Plant Health Inspection Service (APHIS) approved a PI treatment dose of 150 Gy for in-country trade in papayas from Hawaii [11].

In 1992, a cobalt-60 facility was completed in Mulberry, Florida, to irradiate grapefruits as a phytosanitary treatment against Caribbean fruit fly to replace ethylene dibromide, which was being banned, making it the first irradiation facility in the world built expressly for PI [12]. However, the facility was not used for PI until several years later, and it did other types of food irradiation. Presently it does not do PI.

The year 1995 marks the start of continuous and growing use of PI as a commercial treatment. In 1994 personnel from the University of Hawaii and Hawaii Department of Agriculture petitioned the US Animal and Plant Health Inspection Service (APHIS) for a limited use permit to allow untreated papayas to be air-freighted to a cobalt-60 irradiation facility in Morton Grove, Illinois, for PI at a minimum dose of 250 Gy. The dose was raised from 150 Gy because research in the literature called into question whether the lower dose was efficacious [13]. APHIS granted that request in early 1995, and on 5 April 1995 the first shipment was made. Over the next five years increasing amounts and types of Hawaiian fruits were shipped to three facilities in the US states of Illinois and New Jersey for distribution in 15 US states and Washington DC [10].

In 1999, the Florida facility built seven years earlier began to be used for PI when guavas from southern Florida were irradiated to control Caribbean fruit fly and shipped to Texas and California. Several other fruits were also commercially irradiated there. In 2000, the facility began treating white-fleshed sweet potato for shipment to California [14]. This was the first use of PI expressly for a quarantine pest that may occur in the adult stage (sweet potato weevil) on shipped commodities and represents a major step in acceptance of PI because plant protection organizations are especially concerned about having live, albeit reproductively sterile, adults on imported commodities.

In 2000, a commercial X-ray facility exclusively designed for PI began operating in Hawaii and brought about the end to shipments of fruit to the mainland USA for irradiation. Until 2010 it comprised the largest use of PI, irradiating almost 4000 tons of sweet potato and fruit per year. In 2013 another PI facility opened in Hawaii, this one using cobalt-60. The first international shipment of commodity irradiated for phytosanitary purposes was mangoes from Australia to New Zealand in December 2004. In 2014, >2000 tons of mangoes and a few other fruits were irradiated. Since 2010, the largest volume of fresh produce irradiated has been in Mexico, and that volume has more than doubled in the last five years to >13,000 tons today. At present, there are 12 irradiation facilities in seven countries doing PI, and they treated >22,000 tons in 2014. The number of facilities and volumes are expected to continue to increase for the foreseeable future.

### 3.1. Generic Phytosanitary Irradiation Treatments Used Commercially

A generic phytosanitary treatment is one specific dose that is used for a group of quarantine pests and/or commodities although not all were tested for efficacy [15]. Although used to a very limited extent with some phytosanitary treatments, the concept has found broad commercial application in PI in that virtually all commercial PI is done using generic doses.

The most used generic dose is a minimum of 400 Gy for all insects except pupae and adults of the order Lepidoptera (moths and butterflies). It is approved for all products exported to the USA. New Zealand has a similar generic dose of 400 Gy for all insects except adult and pupae of Lepidoptera [16], but it includes mites of the family Tetranychidae (spider mites). Furthermore it is prohibited for use on disease vectoring species, because, although efficacious to prevent further development or reproduction of the insects, 400 Gy has not been shown to prevent disease transmission before the insect vectors die. However, the 400 Gy dose approved by New Zealand is only for mangoes, lychees, tomatoes, and capsicum peppers. A generic dose of 500 Gy is approved in New Zealand for mites other than Tetranychidae.

A generic dose of 250 Gy for import of lychee and mango into New Zealand is approved against regulated pests including a wide variety of species from the insect orders Coleoptera (beetles), Diptera (flies), Hemiptera (scales, mealybugs, whiteflies, among others), Lepidoptera, and Thysanoptera (thrips). A generic 300 Gy dose for Australian mangoes exported to Malaysia includes many of the same regulated pests for New Zealand, but also includes the mango seed weevil. The reason that the dose is 300 Gy for Malaysia instead of 250 Gy is because the mango seed weevil can become established in Malaysia while mangoes are not grown in New Zealand. Hallman [15] discusses why the dose to disinfest mangoes of seed weevil was set at 300 Gy and not lower. A dose of 150 Gy against all weevils has recently been recommended [17].

Tephritid fruit flies are the most important group of regulated pests requiring a phytosanitary treatment in fresh fruits because of their ubiquity, cryptic nature while infesting fruit, and broad host range [18]. A generic dose of 150 Gy against fruit flies of the family Tephritidae (e.g., Mediterranean fruit fly, oriental fruit fly) is broadly accepted and is commercially used in a few instances where the only regulated pests in certain fruits are these flies [15].

### 3.2. New Generic Phytosanitary Irradiation Treatments Proposed

A number of new generic doses have recently been proposed (Table 1). A review of literature concluded that 400 Gy would suffice for pupae of Lepidoptera with a measure of efficacy being prevention of hatch of eggs laid by moths emerging from irradiated pupae [19]. This treatment dose was proposed to the International Plant Protection Convention (IPPC) for inclusion in the phytosanitary treatment manual [20], but was rejected for lack of sufficient large-scale tests with tens of thousands of insects. Nevertheless, although the IPPC also rejected a proposed dose of 400 Gy against all insects except pupae and adults of Lepidoptera, that dose is the one used for ~95% of all commodities irradiated for phytosanitary purposes.

Likewise, a dose of 250 Gy proposed for use against all eggs and larvae of Lepidoptera [21] with the measure of efficacy being prevention of normal-looking adults was rejected by the IPPC for lack of sufficient large-scale testing. However, there is sufficient large-scale testing for the most important family in Lepidoptera, the Tortricidae (e.g., codling moth, oriental fruit moth, light brown apple moth), that would satisfy the requirements of the IPPC.

Although a generic PI treatment for all Tephritidae of 150 Gy is broadly accepted, lower doses for individual species allow for use in specific cases. For example, guavas from Florida may be irradiated with 70 Gy against Caribbean fruit fly and shipped to other parts of the USA. Generic doses for groups of pest species within the family Tephritidae may be justifiable; a generic dose of 70 Gy has been proposed for all fruits infested with species of the tephritid genus *Anastrepha*, which includes all but a few of the species attacking commercial fruit in the American tropics and subtropics [22]. That dose could be used on mangoes exported from Mexico to the USA. Currently almost all mangoes exported to the USA from Mexico are treated with water at 46.1 °C, but irradiation provides for a safer and better quality mango [23].

After tephritid fruit flies and Lepidoptera, the next most important group of regulated pests on fresh fruits and vegetables is mealybugs, which are general surface pests on many fruits and vegetables [1]. A generic dose of 250 Gy supported by large scale studies with several species has been proposed [24].

A generic dose of 150 Gy is proposed for weevils of the main weevil family Curculionidae [17]. This dose would allow mangoes from areas that have one of more of the weevils that infest mangoes to be treated with 150 Gy instead of 300 or 400 Gy.

Hallman *et al.* [25] conclude that the generic doses of 400 and 500 Gy, respectively, for Tetranychidae and all other mites accepted by Australia and New Zealand [16], although not supported by large-scale confirmatory testing, are probably high enough to be phytosanitarily safe. After large-scale confirmatory testing it might be possible to develop somewhat lower doses.

Hallman [26] argues that the 400 Gy generic dose for insects other than pupa and adult Lepidoptera can be lowered to 300 Gy with minimal increase in phytosanitary risk. This dose would still leave a respectable margin of security because research indicates that these insects can be controlled with ~250 Gy. Data from this study also indicate that doses of ~250 and 200 Gy, respectively, might suffice for armoured scales (family Diaspididae) and leafminers (family Agromyzidae).

**Table 1:** Generic phytosanitary irradiation doses approved or proposed.

Pest Group	Dose (Gy)	Approved and Used	References
Tephritidae (fruit flies)	150	yes	[9,27]
Insects except pupal and adult Lepidoptera for export to USA	400	yes	[27]
Insects and Tetranychidae (spider mites) except pupal and adult Lepidoptera or disease vectors for export to New Zealand	400	yes	[16]
Mites other than Tetranychidae	500	yes	[16,25]
Pests of lychee and mango to New Zealand	250	yes	[16]
Pests of mango to Malaysia	300	yes	[16]
Pupae of Lepidoptera	400	no	[19]
Eggs and larvae of Lepidoptera	250	no	[21]
Eggs and larvae of the lepidopterous family Tortricidae	250	no	[21]
Fruit flies of the genus *Anastrepha*	70	no	[22]
Mealybugs	250	no	[24]
Weevils	150	no	[17]
Insects except pupal and adult Lepidoptera	300	no	[26]

## 4. Effect of Phytosanitary Irradiation on Fruits and Vegetables

For phytosanitary treatments to be commercially feasible they must be efficacious and commodities must not be rendered unmarketable by them. At the doses used for PI (70 to 400 Gy), more fresh fruits and vegetables tolerate radiation than any other broadly applicable commercial treatment [2]. Applying radiation to standard packed pallet loads is economical because commodity can be treated with minimum handling; however, this may result in much of the load receiving a much greater radiation dose than necessary to ensure that all parts of the load receive at least the minimum required dose. For example, fruits on the outside of pallet loads irradiated at a cobalt-60 facility in South Africa can receive almost four times the minimum prescribed dose to ensure that the minimum dose is absorbed by the entire load [28]. This is for two reasons: (1) the irradiation facility aims to deliver a minimum dose higher than the prescribed PI treatment dose to ensure that the actual minimum dose received is at least the PI treatment dose within a large degree of statistical confidence; and (2) the dose uniformity ratio for the process (the ratio of maximum to minimum absorbed dose in the production lot) may be as high as three. Electron beams cannot penetrate deeply and therefore these facilities are designed to process boxes of product and not pallets, and can achieve a dose uniformity ratio as low as ~1.2. No matter what source of radiation is used, commodities in their commercial packaging should be tested for radiotolerance at the maximum doses that could be absorbed by any part of the load configuration.

It may also be necessary to adapt harvesting procedures when using PI. Radiation may slow the rate of fruit ripening, and fruit that is commercially harvested before it is ready to be eaten, such as papayas, mangoes, and many other tropical fruits, may not ripen as quickly as non-irradiated fruit. Delaying the physiological stage of harvest may improve ripening quality, and delayed harvest alone should result in improved quality. However, the fact that papaya, mango, and guava have been irradiated commercially from different countries for many years while in the mature hard green stage testifies to the ability of fruit to tolerate PI and still ripen normally.

The International Database on Insect Disinfestation and Sterilization maintained by the Joint FAO/IAEA Programme on Nuclear Techniques in Food and Agriculture is developing an annotated database on tolerance of fresh commodities to ionizing radiation [29]. This database will help facilitate international use of the technology as it can be used by industry to determine the feasibility of using commercial PI. A publication summarizing the literature with a discussion of research methodology and further research that might be warranted is also planned.

PI has been applied commercially to a wide variety of fresh commodities, and the fact that they have been successfully marketed after receiving doses that may considerably exceed the minimum doses required for efficacy attests to the broad tolerance of fresh commodities at doses of radiation required for phytosanitation. The following commodities are irradiated with minimum doses of 400 Gy that results in much of the commodity absorbing approximately twice that dose without rendering them unmarketable: bell peppers, dragonfruit, grape, grapefruit, guava, lime, longan, lychee, mango, manzano pepper, papaya, persimmon, plum, rambutan, sweet potato, and tomato. A few of the commodities suffered initial problems related to bottlenecks in processing and marketing when the treatments were first being done because of delays in moving the product through the novel marketing channels, but these problems were solved with experience. In time all cases resulted in acceptable product reaching consumers. Often the product was in better condition because irradiation resulted in a better quality product than alternative treatments. Other fruits, such as rambutan, could not be successfully marketed until PI became available.

## 5. Future Needs

The use of PI to enable trade in quarantined commodities is increasing annually. More countries are participating in this trade, the number of different commodities traded is growing, increasing volumes of produce are being treated, and PI is being used against a growing number of different quarantine pests. However, traded volumes are still rather modest considering the potential of the technology. To optimize potential, several challenges should be addressed.

### 5.1. Streamline Commercial Approval Process

Currently a number of countries that could benefit from PI do not have the regulations in place to permit it. Others have regulations that are overly complex; for example specifying certain fruits and vegetables that can be treated by PI but requiring additional commodities to undergo comprehensive tests to demonstrate that the process does not significantly alter nutritive quality, even if they are very similar to the ones already approved. Other countries regulate in terms of broad food categories, such as fresh fruits and vegetables, or on the basis of a maximum dose limit applicable to all fresh commodities. There is no technical reason why permissions cannot be given to fresh commodities as broad food categories. Countries are encouraged to use international standards, adapt regulations and approaches that have been successfully developed by other countries and organizations, and consider streamlining the approval of PI.

With increasing use of PI it is more efficient to adopt a multi-lateral approach to trade between several countries or regions affected by specific phytosanitary pests, as exemplified by the International Plant Protection Convention phytosanitary treatment manual [20], instead of relying on individual bilateral agreements with each trading partner.

### 5.2. Reduce Negative Impact of Labeling

Products irradiated for phytosanitary purposes must be labeled as irradiated, which is considered as unnecessarily negative by some because it may be interpreted as a warning. In contrast, products disinfested by fumigation are not labeled although fumigant residues may be found, and mangoes treated by hot water immersion need not be labeled although it is the only treatment documented to have killed consumers of treated product [23]. Knowing what has been done to their food is a right of the consumer but it should be done in a uniform way; if fruit processed by other phytosanitary treatments need not be labeled, fruit irradiated for phytosanitary purposes should not require labeling either.

### 5.3. More Accessible Commercial Application

The start-up costs of a PI facility are often more expensive than for other phytosanitary treatments because of the initial cost of construction. The business is seasonal, and the need to transport product to those facilities and associated logistics can also affect operating costs. Tools to decide on the optimum location and business model for operation would assist commercial siting and use of facilities. Reducing costs of PI may involve development of modular approaches to building facilities so that a simple facility can be expanded by the addition of more capacity as throughput increases or developing tailor made PI facilities with in-line irradiators incorporated into individual packing operations. However, as facilities become smaller and more localized, regulatory challenges in plant protection and safety may need to be considered at each of these localities, thus, resulting in increased relative costs of operation.

### 5.4. Wider Acceptance of Phytosanitary Irradiation

Much has been discussed about consumer and retailer wariness towards food irradiation. The primary issue seems to be fear of consumer rejection among retailers; in practice the indications are that when a retailer makes irradiated food available it is accepted by the majority of consumers and sells well [30]. The refusal of the “organic” industry to accept PI is discussed by Hallman [1] and considered to be in part due simply to a conservative mind-set of adherents to this philosophy that could change in time as PI becomes more familiar. Low-dose irradiation is routinely used to screen for bones in meat and foreign objects in food. The irradiation of commodities (including food) is also used as a security measure and to screen for contraband. These applications of ionizing radiation do not affect the certified organic status of a food, whereas using PI results in that product not being considered “organic”.

### 5.5. Phytosanitary Irradiation Efficacy Doses

**Figure 1:**
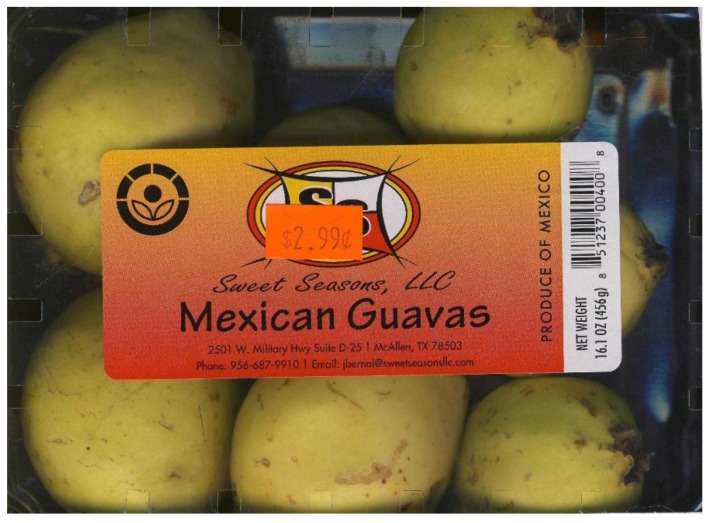
Mexican guava is the largest single use of phytosanitary irradiation. It is successfully irradiated at a minimum dose of 400 Gy to control a wide variety of insects that may infest it including fruit flies, mealybugs, caterpillars, scale insects, weevils, and whiteflies before export to the USA. With appropriate research that dose might be reduced to 250 Gy.

Indications are that most currently accepted commercial applications of PI specify doses that are higher than necessary (Figure 1). Ongoing research throughout the world supports new PI doses and the lowering of existing doses. A special issue of Florida Entomologist reports on much recently finished research from an IAEA Coordinated Research Project that supports various individual and generic PI doses that should be considered by plant protection organizations. Some research has been done and continues to be done using methodology (e.g., artificial infestation) that is not accepted by plant protection organizations. Other research is wasted pursuing objectives that need no further research, such as most radiotolerant stage and studies with insects that have already been adequately studied. Researchers should adopt accepted methods and base research on real needs. A new international phytosanitary measures research group is a good way to coordinate effort [31].

### 5.6. Solve Issues of Factors Affecting Efficacy

Of the various factors that have been hypothesized as affecting the efficacy of PI [32], oxygen content is the one that has already impacted regulation of the treatment. Irradiation under hypoxic conditions may reduce efficacy. Therefore, plant protection organizations have prohibited the use of PI on commodities stored in hypoxic atmospheres if the research supporting its use under hypoxic conditions has not been done [27,33]. Although some research has shown that this is not a problem for tephritid fruit flies [34,35] definitive research is needed to put to rest this issue for this important group of quarantine pests. Additionally, some research was done using larvae reared on diet and inserted into holes bored to the centre of fruits [35], which presents a series of untested assumptions concerning results of these studies. Regardless of the final conclusion for fruit flies, irradiation in hypoxic atmospheres seems more of a concern for most other quarantine pests, which seem to respond to hypoxia to a greater extent than fruit flies [36].

Another issue that is of concern is whether temperature during irradiation affects efficacy. It does not seem to be the case [32], but further research may be warranted before putting the issue to rest.

The two main physical characteristics that differentiate gamma from electron beam irradiation are depth of penetration and dose rate; electron beams have low penetration but deliver very high dose rates whereas gamma rays are penetrating and have relatively low dose rates. Dose rate is hypothesized to directly affect PI efficacy; *i.e.*, a faster dose rate may lead to increased efficacy because it overwhelms radiation damage repair mechanisms. High dose rates also result in oxygen depletion and the presence of oxygen significantly increases the efficiency and nature of radiation damage. Therefore, dose rate effects are especially of interest because electron beams are increasingly being used in PI and most research was previously done with slower gamma sources. Fresh commodities and insect pests could conceivably have different tolerances to fast dose-rate sources but there are few studies in this area and there is no evidence of the effect being significant at the dose rates used commercially.

## 6. Conclusions

The commercial use of phytosanitary irradiation (PI) increases by ~10% annually as the use of chemical fumigants is restricted, irradiation treatment protocols and their applicability to different fruits and vegetables are accepted internationally, and more countries and traders adopt the procedure for intra- and inter-national shipments.

Generic treatments, one dose is used for a group of pests and/or commodities although not all have been tested for efficacy, has found broad commercial application in PI in that virtually all commercial PI is done using them.

Fresh fruits and vegetables tolerate PI better than any other broadly used phytosanitary treatment.

Advances that would help facilitate the favourable use of PI include streamlining the approval process, making the technology more accessible to potential users, lowering and broadening doses, and solving potential issues related to factors that might affect efficacy.
